# Do intravitreal anti-vascular endothelial growth factor agents lead to renal adverse events? A pharmacovigilance real-world study

**DOI:** 10.3389/fmed.2023.1100397

**Published:** 2023-02-14

**Authors:** Lin Jiang, Liying Peng, Yangzhong Zhou, Gang Chen, Bin Zhao, Mingxi Li, Xuemei Li

**Affiliations:** ^1^Pharmacy Department, Peking Union Medical College Hospital, Peking Union Medical College, Chinese Academy of Medical Sciences, Beijing, China; ^2^Nephrology Department, Peking Union Medical College Hospital, Peking Union Medical College, Chinese Academy of Medical Sciences, Beijing, China

**Keywords:** acute kidney injury, vascular endothelial growth factor, adverse event reporting system, pharmacovigilance, ophthalmic medicine

## Abstract

**Purpose:**

Intravitreal vascular endothelial growth factor (VEGF) blockade is essential in many macular edema diseases treatment. However, intravitreal VEGF treatment has been reported to lead to deteriorated proteinuria and renal function. This study aimed to explore the relationship between renal adverse events (AEs) and the intravitreal use of VEGF inhibitors.

**Method:**

In the FDA’s Adverse Event Reporting System (FAERS) database, we searched for renal AEs of patients receiving various anti-VEGF drugs. We performed statistics on renal AEs in patients treated with Aflibercept, Bevacizumab, Ranibizumab, and Brolucizumab (from January 2004 to September 2022) using disproportionate and Bayesian analysis. We also investigated the time to onset, fatality, and hospitalization rates of renal AEs.

**Results:**

We identified 80 reports. Renal AEs were most frequently associated with Ranibizumab (46.25%) and Aflibercept (42.50%). However, the association between intravitreal anti-VEGFs and renal AEs was insignificant since the reporting odds ratio of Aflibercept, Bevacizumab, Ranibizumab, and Brolucizumab were 0.23 (0.16, 0.32), 0.24 (0.11, 0.49), 0.37 (0.27, 0.51) and 0.15 (0.04, 0.61), respectively. The median time to renal AEs onsets was 37.5 (interquartile range 11.0–107.3) days. The hospitalization and fatality rates in patients who developed renal AEs were 40.24 and 9.76%, respectively.

**Conclusion:**

There are no clear signals for the risk of renal AEs following various intravitreal anti-VEGF drugs based on FARES data.

## 1. Background

The systemic administration of vascular endothelial growth factor (VEGF) inhibiting monoclonal antibodies has been applied in oncology to inhibit angiogenesis in varied neoplasms since the 1990s (1). The United States Food and Drug Administration (FDA) has approved several types of anti-VEGF agents, including Bevacizumab (Avastin®, 2004), Pegaptanib (Macugen®, 2004), Ranibizumab (Lucentis®,2006), Aflibercept (Zaltrap®; Eylea®, 2011), and Brolucizumab (Beovu®, 2019). Nowadays, the clinical use of anti-VEGF agents has expanded to intravitreal treatment since angiogenesis is essential for the progression of ophthalmic diseases (2). Evidence shows that VEGF injections are effective in clinical trials involving several types of retinal vascular pathology and ocular neovascularization (3, 4). After 2000, Aflibercept, Ranibizumab, and Pegaptanib received approvals for indications like proliferative diabetic retinopathy (DR), diabetic macular edema (DME), age-related macular degeneration (AMD), and retinal vein occlusion (RVO) (5). Bevacizumab and Brolucizumab have also been used off-label for vascular-related ophthalmic diseases (6).

It has been widely recognized that systemically administrated anti-VEGF agents are linked to increased risks of cardiovascular events (7) and renal adverse events (AEs), including proteinuria, acute kidney injury (AKI), glomerular disease, and thrombotic microangiopathy (TMA) (8–10). For intravitreal anti-VEGF administrations, the dose usually ranged from 1/150 to 1/400 of that in systemic use (11–13). However, systemic absorption is noted after intravitreal use (14). One post-marketing study informed that the anti-VEGF-related severe AEs were beyond expectations when applied in an ophthalmology setting (15). Renal AEs after intravitreal anti-VEGFs have emerged in clinical cases (12, 16, 17) and have been listed in meta-analysis (18, 19).

As a complement to clinical trials, post-marketing AE monitoring is essential to expand our understanding of the potential renal AEs of intravitreal anti-VEGFs. Unfortunately, except for intravitreal anti-VEGFs (15), there were limited pharmacovigilance studies on other aspects of systemic safety. Therefore, renal AEs have been neglected. Knowledge of the detailed safety profile of renal AEs following intravitreal anti-VEGF regimens in real-world clinical practice is lacking. Therefore, we aimed to evaluate the links between various intravitreal anti-VEGF agents and renal AEs in a real-world setting based on the FDA’s Adverse Event Reporting System (FAERS) until September 2022. The FAERS database contains adverse event reports, medication error reports, and product quality complaints resulting in adverse events that were submitted to FDA. Furthermore, we examined the time to onset, fatality rate, and hospitalization rate for renal AEs following intravitreal anti-VEGF regimens.

## 2. Methods

### 2.1. Data source

We performed a retrospective pharmacovigilance study using data from the FAERS database between January 2004 and September 2022. The FAERS is a public spontaneous reporting system (SRS) that contains information about adverse drug events provided by global health professionals, patients, and manufacturers. FAERS data files describe demographic and administrative information (DEMO), drug information (DRUG), preferred terms (PTs) coded for the adverse events (REAC), patient outcomes (OUTC), report sources (RPSR), therapy start dates, and end dates for reported drugs (THER), and indications for drug administration (INDI).

**Figure 1:**
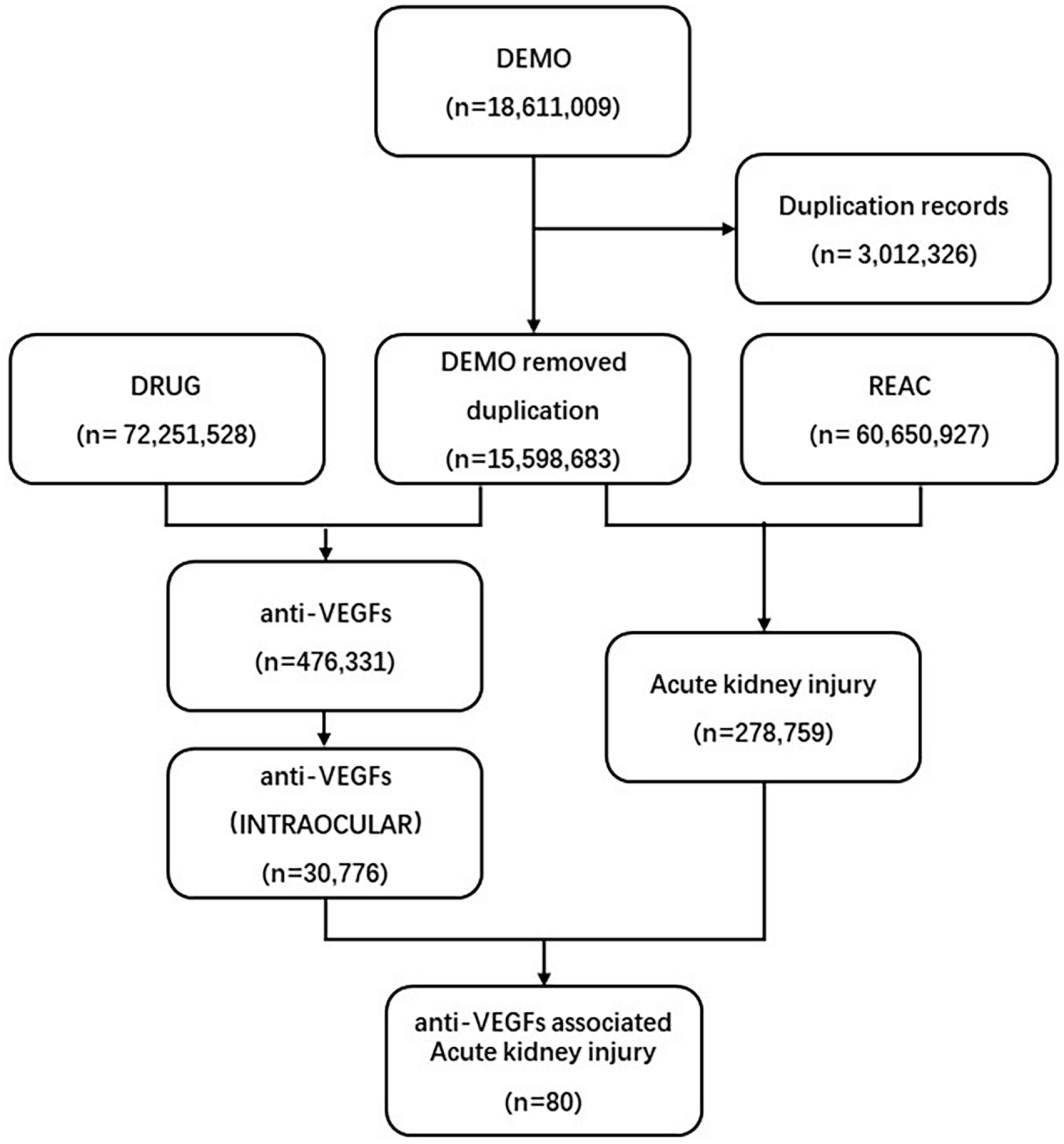
Process of the selection of cases of intravitreal anti-VEGF-associated renal adverse effects from the FAERS database. VEGF, vascular endothelial growth factor; FAERS, Food and Drug Administration’s Adverse Event Reporting System.

We screened 18,611,009 reports from the FAERS database. We removed duplicated records by selecting the latest FDA_DT (Date FDA received Case) when the CASEID (Number for identifying a FAERS case) and FDA_DT were the same. Finally, we included 15,598,683 reports for further analysis (Figure 1).

### 2.2. Data mapping

We investigated the REAC files for the comprehensive Medical Dictionary for Regulatory Activities (MedDRA v23.1). MedDRA defined terms related to renal AEs as follows: “acute kidney injury,” “subacute kidney injury,” “kidney failure,” “oliguria,” “anuria,” “dialysis,” “proteinuria,” “hematuria,” “blood creatinine increased,” “blood urea increased,” “nephritis,” “nephritis toxic,” “tubulointerstitial nephritis,” “renal tubular injury,” “glomerulonephritis acute,” “glomerulonephritis rapid progressive,” “autoimmune nephritis,” “glomerulonephritis membranous,” “glomerulonephritis minimal lesion,” “glomerulonephritis membranoproliferative,” “glomerulonephritis proliferative,” “nephritic syndrome,” “thrombotic microangiopathy.” We chose generic and brand names of anti-VEGF regimes by utilizing the MICROMEDEX (Index Nominum) as a dictionary in the data mining process. The assessment considered drugs recorded as “Primary Suspect” or “Secondary Suspect” (PS an SS in role code field) and routed as “INTRAVITREAL.”

### 2.3. Data mining

Based on the rationale of disproportionality analysis and Bayesian analysis, we employed the reporting odds ratio (ROR), the proportional reporting ratio (PRR), the Bayesian confidence propagation neural network (BCPNN), and the multi-item gamma Poisson shrinker (MGPS) algorithms to investigate the associations between the drug and the given AEs. The equations and criteria for the four algorithms are listed in Table 1.

**Table 1:** Summary of major algorithms used for signal detection.

Algorithms	Equation*	Criteria
ROR	ROR = (a/b)/(c/d)	95% CI > 1, *N* ≥ 2
	95%CI = e^ln(ROR) ± 1.96(1/a + 1/b + 1/c + 1/d)^0.5^	
PRR	PRR = (a/(a + c))/(b/(b + d))	PRR ≥ 2, *χ*^2^ ≥ 4, *N* ≥ 3
	*χ*^2^ = Σ((O-E)^2^/E); (O = a, E = (a + b)(a + c)/(a + b + c + d))	
BCPNN	IC = log_2_a(a + b + c + d)/((a + c)(a + b))	IC025 > 0
	IC025 = e^ln(IC)-1.96(1/a + 1/b + 1/c + 1/d)^0.5^	
MGPS	EBGM = a(a + b + c + d)/((a + c)(a + b))	EBGM05 > 2, *N* > 0
	EBGM05 = e^ln(EBGM)-1.64(1/a + 1/b + 1/c + 1/d)^0.5^	

*a: number of reports containing both the suspect drug and the suspect adverse drug reaction. b: number of reports containing the suspect adverse drug reaction with other medications (except the drug of interest). c: number of reports containing the suspect drug with other adverse drug reactions (except the event of interest). d: number of reports containing other medications and other adverse drug reactions. ROR, reporting odds ratio; CI, confidence interval; N, the number of co-occurrences; PRR, proportional reporting ratio; *χ*^2^, Chi-squared; BCPNN, Bayesian confidence propagation neural network; IC, information component; IC025, the lower limit of the 95% two-sided CI of the IC; MGPS, multi-item gamma Poisson shrinker; EBGM, empirical Bayesian geometric mean; EBGM05, the lower 90% one-sided CI of EBGM.

We compared the associations between renal AEs and different anti-VEGF agents. We also evaluated the time to onset of renal AEs for different intravitreal anti-VEGF agents, defined as the interval between the EVENT_DT (adverse event onset date) and the START_DT (start date of the intravitreal anti-VEGF administration). The records with incorrect entries or erred input (EVETN_DT earlier than START_DT) and duplicate reports were excluded. Additionally, we analyzed reports of fatal events due to adverse drug reactions and calculated the fatality rate by dividing the catastrophic events by the total number of occurrences of intravitreal anti-VEGF-induced renal AEs.

### 2.4. Statistical analysis

We used descriptive analysis to summarize the clinical features of the patients with renal AEs resulting from intravitreal administration of anti-VEGFs in the FAERS database. The time to onset of renal AEs among different anti-VEGFs was compared using non-parametric tests (the Mann–Whitney test for dichotomous variables and the Kruskal–Wallis test when there were more than two subgroups of respondents). Pearson’s Chi-square or Fisher’s exact test was used to compare the fatality rates between different anti-VEGFs. We set statistical significance at *p < 0.05* with 95% confidence intervals. Data mining and statistical analysis were performed by SAS, version 9.4 (SAS Institute Inc., Cary, NC, United States).

## 3. Results

### 3.1. Descriptive analysis

A total number of 30,776 AEs related to intravitreal administration of anti-VEGFs and 278,759 renal AEs were documented in the FAERS database dated from January 2004 to September 2022 (Figure 1). We merged the signals above and finally screened 80 renal AE reports suspected of intravitreal administration of anti-VEGFs and summarized the clinical features of these patients in Table 2. The case numbers were comparable in North America (36.25%) and Europe (40.00%). Healthcare professionals reported 67.50% of the cases. The morbidity seemed to be equal between males (29/50, 58.0%) and females (21/50, 42.0%). The average age for all patients was 70.86 (±11.78) years, and we found no age difference between affected males and females (*p* = 0.671). Most of the affected patients were elderly (>65-year-old, 73.81%) and middle-aged (45–64 years old, 21.43%). The renal AEs related to intravitreal anti-VEGFs were most frequently associated with Ranibizumab (46.25%) and Aflibercept (42.50%). Intravitreal pegaptanib has not been reported with renal AEs in the current FAERS database. Among the renal AEs, anti-VEGFs were dominantly administrated in AMD (50.00%), DRE (20.00%), and DR (10.00%).

**Table 2:** Clinical characteristics of patients with renal AEs after intraocular administration of anti-VEGFs sourced from the FAERS database (January 2004 to September 2022).

Characteristics	Reports, no. (%)
**Reporting region**	
North America	29 (36.25)
Europe	32 (40.00)
Asia	12 (15.00)
Oceania	4 (5.00)
South America	3 (3.75)
Africa	0 (0.00)
**Reporters**	
Health-care professionals	54 (67.50)
Non-health-care professionals	15 (18.75)
Unspecified	11 (13.75)
**Reporting year**	
2022 (Until September)	1 (1.25)
2021	5 (6.25)
2020	9 (11.25)
2019	6 (7.50)
2018	4 (5.00)
2017	17 (21.25)
2016	8 (10.00)
2015	7 (8.75)
2014	7 (8.75)
2013	0 (0.00)
2012	2 (2.50)
2011	6 (7.50)
2010	3 (3.75)
2009	5 (6.25)
**Sex**	
Male	29/50 (58.0)
Female	21/50 (42.0)
**Age (years)**	
<18	0 (0.00)
18–44	2/42 (4.76)
45–64	9/42 (21.43)
>65	31/42 (73.81)
Unknown or missing	38/80 (47.50)
**Intraocular anti-VEGFs as suspected drugs**	
Aflibercetp	34 (42.50)
Bevacizumab	7 (8.75)
Ranibizumab	37 (46.25)
Brolucizumab	2 (2.50)
**Indications**	
Age-related macular degeneration	40 (50.00)
Diabetic retinal edema	16 (20.00)
Diabetic retinopathy	8 (10.00)
Retinal vein occlusion	2 (2.50)
Retinal neovascularization	1 (1.25)
Neovascular glaucoma	1 (1.25)
Choroidal neovascularization	1 (1.25)
Retinal detachment	1 (1.25)
Unknown or missing indications	10 (12.50)

VEGF: vascular endothelial growth factor; AE: adverse event; FAERS: Food and Drug Administration’s Adverse Event Reporting System.

### 3.2. Disproportionality analysis and Bayesian analysis

Based on the four algorithms’ criteria, we detected renal AEs signals for different anti-VEGFs for intravitreal administration and listed the results in Table 3. All anti-VEGFs showed insignificant associations with renal AEs due to their weak ROR, PRR, IC025, and empirical Bayes geometric mean (EBGM) values. The ROR of Aflibercept, Bevacizumab, Ranibizumab, and Brolucizumab were 0.23 (0.16, 0.32), 0.24 (0.11, 0.49), 0.37 (0.27, 0.51) and 0.15 (0.04, 0.61), respectively.

**Table 3:** Association of different intraocular anti-VEGF regimens with renal AEs.

Drug	*N*	ROR (95% two-sided CI)	PRR (*χ*^2^)	IC (IC025)	EBGM (EBGM05)
Aflibercept	34	0.23 (0.16, 0.32)	0.23 (86.9)	−2.1 (0.00)	0.23 (0.18)
Bevacizumab	7	0.24 (0.11, 0.49)	0.24 (17.31)	−2.07 (0.00)	0.24 (0.13)
Ranibizumab	37	0.37 (0.27, 0.51)	0.37 (39.28)	−1.42 (0.00)	0.37 (0.29)
Brolucizumab	2	0.15 (0.04, 0.61)	0.15 (9.37)	−2.69 (0.00)	0.15 (0.05)

VEGF: vascular endothelial growth factor; AE: adverse event; N: the number of reports of intraocular anti-VEGF-associated renal AEs; ROR: reporting odds ratio; CI: confidence interval; PRR: proportional reporting ratio; *χ*^2^: Chi-squared; IC: information component; EBGM: empirical Bayes geometric mean.

### 3.3. Time to onset of renal AEs associated with intravitreal anti-VEGFs

**Figure 2:**
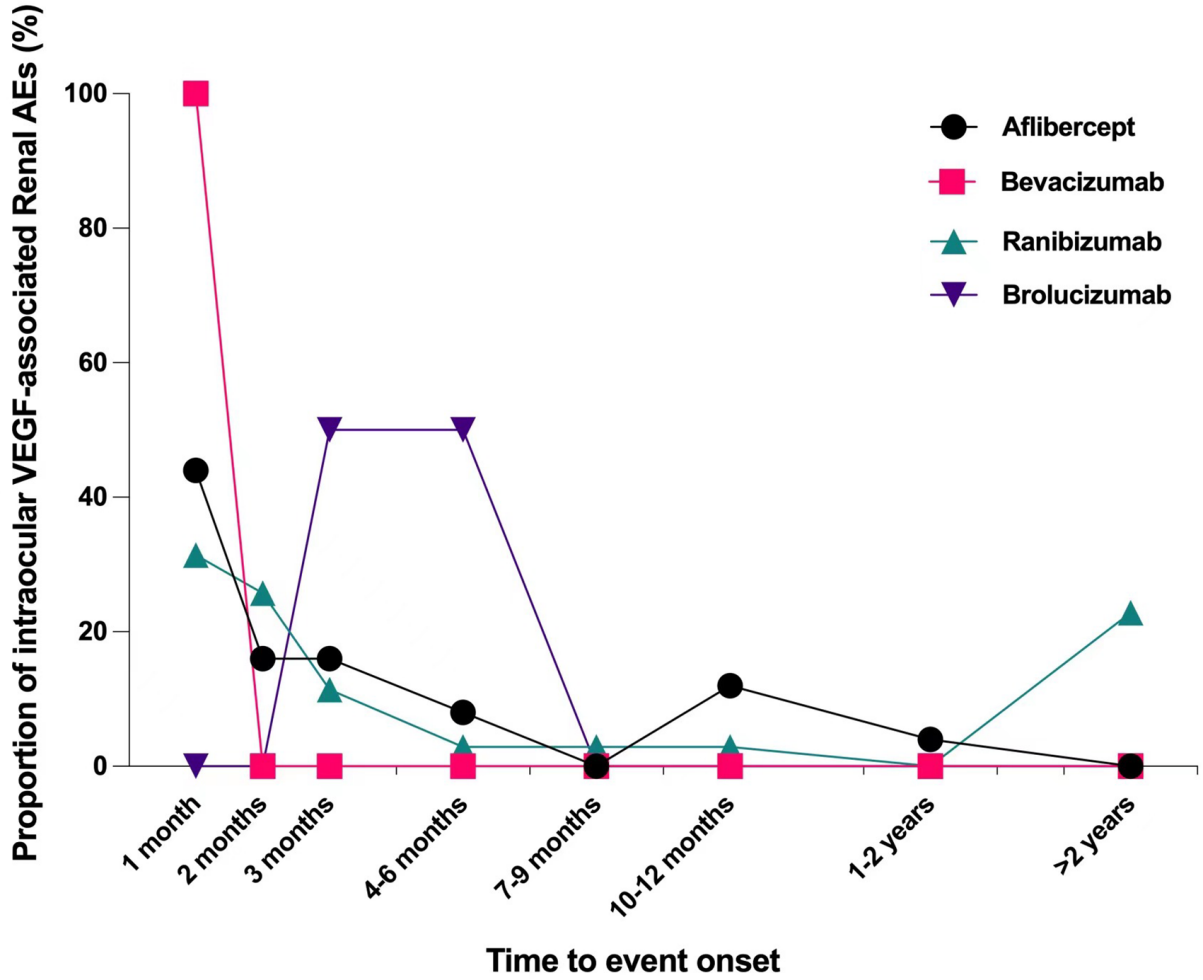
Time to event onset of renal adverse effects following intravitreal administration of anti-VEGF agents. VEGF, vascular endothelial growth factor.

Overall, the median time to onset renal AEs associated with intravitreal anti-VEGFs was 37.5 days (interquartile range [IQR] 11.0–107.3 days) after administering drugs. The times to onset of renal AEs for each intravitreal anti-VEGF regimen was described in Figure 2. Near 40% of the renal AEs occurred in the first month (37.5%), and more than half (57.81%) occurred in the first 2 months. Noteworthily, we found that 12.5% of the renal AEs could happen as soon as the first dose of intravitreal anti-VEGF administration. Kruskal–Wallis test detected no significant difference in time to onset of renal AEs among different anti-VEGFs (*p* = 0.492).

### 3.4. Prognosis due to intravitreal anti-VEGF-associated renal AEs

To analyze the prognosis of renal AEs associated with intravitreal anti-VEGFs, we assessed the fatality and hospitalization rate (initial or prolonged) due to renal AEs following Aflibercept, Bevacizumab, Ranibizumab, and Brolucizumab in the FAERS database (Table 4). Generally, the hospitalization rate of intravitreal anti-VEGF-associated renal AEs was 40.24%, the life-threatening events rate was 8.54%, and the fatality rate of 9.76%. There was no significant difference in hospitalization rates (*p* = 0.693), life-threatening rates (*p* = 0.758), and fatality rates (*p* = 0.630) across different intravitreal anti-VEGFs (Pearson’s Chi-square test for overall comparison).

**Table 4:** Outcomes after intraocular VEGF-associated renal AEs.

	Aflibercept	Bevacizumab	Ranibizumab	Brolucizumab	Total
Death	3 (8.57)	0 (0)	5 (13.51)	0 (0)	8 (9.76)
Disabled	1 (2.86)	0 (0)	0 (0)	1 (33.33)	2 (2.44)
Hospitalization	15 (42.86)	2 (28.57)	14 (37.84)	2 (66.67)	33 (40.24)
Life-threatening	2 (5.71)	1 (14.29)	4 (10.81)	0 (0)	7 (8.54)
Other serious	14 (40.00)	4 (57.14)	14 (37.84)	0 (0)	32 (39.02)

VEGF: vascular endothelial growth factor; AE: adverse event.

## 4. Discussion

In this study, we completed the first collection until recently to seek confirmation of renal AEs after intravitreal anti-VEGF agents based on the FAERS pharmacovigilance real-world practice. Interestingly, although we found 80 renal AE reports in the database, all four members of intravitreal anti-VEGFs demonstrated little association with renal AEs by signal detection algorithms.

Our findings were consistent with some studies and reports that focused on endothelial toxicity and renal damage after intravitreal anti-VEGF. A retrospective cohort in Japan, which included 69 diabetic patients with DR, showed no significant increase in creatinine 7–30 days after applying intravitreal Bevacizumab, Aflibercept, or Ranibizumab (20). Another retrospective review of 85 patients with DME suggested that regular intravitreal VEGF inhibition did not induce increased proteinuria or affect kidney function over a mean duration of 2.6 years (21). A recent randomized control trial that enrolled 660 DME patients revealed no significant change in proteinuria after intravitreal VEGFs for up to 52 weeks (22).

Our pharmacovigilance analysis echoed such clinical studies and detected no association between intravitreal anti-VEGFs and renal AEs. In the scenario of a rare and potential AE issue, clinical cohorts and trials are far from convincing to draw a definitive conclusion due to their strict inclusion criteria, limited sample sizes, and relatively short observation periods. The SRS could be a fitted source for new evidence.

However, the relationship between systemic damages and local anti-VEGF injections is still controversial (12, 16–19). Some mechanisms of anti-VEGF drugs predispose the potential to develop renal AEs. VEGF performs specific effects on vascular endothelial cells. It is believed that the VEGF-driven results on neovascularization are essential for the reperfusion of ischemic tissues. The anti-VEGF drugs may increase the risk of cardiovascular and renal AEs (7–10, 23–26). The anti-VEGF agents interrupt the podocyte-endothelial VEGF signaling axis, resulting in decreased glomerular capillary endothelial cell permeability, reduced endothelial cell proliferation, and podocyte detachment (27). Meanwhile, anti-VEGF effects decrease nephrin expression in glomeruli, leading to the detachment and atrophy of endothelial cells (28). Though far lower dose than systemic administration, the intravitreal injection of anti-VEGFs still results in detectable serum levels (14, 29) and glomeruli bindings (30), then consequently leads to systemic VEGF inhibition (14, 29). Proteinuria (16) and TMA (12) cases after intravitreal VEGF inhibitors hinted at the possible side effects on podocytes and endothelial cells.

Analyzing from another perspective, we cannot completely exclude the possibility of the systemic damages induced by local anti-VEGF injections. Interestingly, we noticed that Pegaptanib resulted in zero renal AEs among four members of intravitreal anti-VEGFs. The possibly fewer clinical application would be a convenient explanation, but Pegaptanib possesses properties that distinguish it from other intravitreal anti-VEGFs. Other than antibodies, Pegaptanib is a ribonucleic acid aptamer that binds to VEGF isoform (1). It is pharmacokinetically short-acting, and its systemic absorption is limited when used intravitreally (1); such characteristics may contribute to the rarity of renal AEs related to intravitreal pegaptanib. On the other hand, we found that Aflibercept resulted in more intravitreal anti-VEGF-associated renal AE reports than Bevacizumab in the current FAERS database (34/80 cases vs. 7/80 cases). This was consistent with a previous finding that Aflibercept was more potent than Bevacizumab in systemic VEGF inhibition after intravitreal injection (29).

Although we spotted some renal AE reports after intravitreal anti-VEGFs in the FARES database, we trusted the accuracy of the renal AEs because healthcare professionals contributed most of the reports. Still, the causality could not be set up due to the negative signals in ROR, PRR, IC, and EBGM for all four anti-VEGFs. No evidence has indicated that renal involvements like proteinuria or decreased glomerular filtration rate occurred more frequently in patients after intravitreal anti-VEGF injections. Additionally, the discrepancies in clinical observations (12, 16–22) also raised the possibility that genetic background might contribute to patients’ susceptibility to renal toxicity.

Based on the above analysis, we should admit that there has been no concrete evidence to prove intravitreal anti-VEGF-associated renal AEs. However, we should keep in mind that this side effect could be possible in elderly diabetic patients.

Once they happened, drug-associated renal AEs in diabetic groups would be harmful. Our data indicated that the hospitalization rate was as high as 31.25% in patients who developed renal AEs after intravitreal anti-VEGFs, and the related fatality rate reached an unneglectable 6.25%. The median time to renal AEs onsets after overall intravitreal anti-VEGFs was 37.5 days, and more than half of the insulted cases were reported to occur within 2 months. Therefore, we should carefully monitor the potential renal AEs in elderly patients during the early administration of intravitreal anti-VEGFs. It was noted that immediate renal AEs could occur in around 10% of all affected patients. Based on current findings, it is not trivial to consider tracing the changes in kidney function in patients who tend to develop AKI.

We acknowledge that our study has some limitations. First, unlike researchers who use standardized data collection methods to report AEs in clinical trials, the FAERS database has inherent limitations in reporting form, such as under-reporting, false, incomplete, inaccurate, and arbitrary reporting. Second, because we lack the total number of patients receiving treatment, we cannot calculate some statistics, such as the incidence of each suspect drug, so signals from the spontaneous reporting system can only be used for qualitative studies. It is also difficult to control for confounding factors such as baseline renal insufficiency, pre-existing kidney disease and comorbidities, and renal complications due to diabetes itself that may influence renal AEs due to the lack of sufficient information. Therefore, a definitive causal relationship between anti-VEGF agents and renal AEs cannot be accurately inferred. Third, accurate dosages for patients are not accurately available from the FARES database, making it impossible to analyze the timing or total dosages of different types of antivascular drugs.

## 5. Conclusion

In this study, we utilized the FAERS database and identified no clear signals for renal AEs following various intravitreal VEGFs in real-world practice. Based on FARES data, it is not possible to infer that local anti-VEGF drug injection causes renal AEs, contrary to some previous case reports. Our findings pave the way for the following pharmacovigilance investigation. We recommend accessing renal AEs and other systemic damages as the primary outcomes in high-quality clinical trials and real-world studies to explore the relationship between intravitreal anti-VEGFs and renal AEs.

## Data availability statement

All necessary data have been presented as tables and figures in the manuscript. Related information is accessible under request to the corresponding author.

## Author contributions

GC and LJ designed the study, analyzed and interpreted data, generated figures and tables, and drafted the manuscript. LP and YZ contributed to manuscript drafting. BZ designed the study and directed the data mining in the FAERS database. ML and XL reviewed and corrected the manuscript. All authors contributed to the article and approved the submitted version.

## Funding

This work was supported by the Sansheng Yeehong TCP Research Foundation (GC), Bethune Charitable Foundation (J202103E006) (GC), and National High-Level Hospital Clinical Research Funding [2022-PUMCH-B-021].

## Conflict of interest

The authors declare that the research was conducted in the absence of any commercial or financial relationships that could be construed as a potential conflict of interest.

## Publisher’s note

All claims expressed in this article are solely those of the authors and do not necessarily represent those of their affiliated organizations, or those of the publisher, the editors and the reviewers. Any product that may be evaluated in this article, or claim that may be made by its manufacturer, is not guaranteed or endorsed by the publisher.

## Abbreviations

AKI: acute kidney injury
VEGF: vascular endothelial growth factor
AE: adverse event
FAERS: Food and Drug Administration’s Adverse Event Reporting System
ROR: reporting odds ratio
PRR: proportional reporting ratio
EBGM: empirical Bayes geometric mean; IQR, interquartile range
FDA: the Food and Drug Administration
SRS: spontaneous reporting system
BCPNN: Bayesian confidence propagation neural network
MGPS: multi-item gamma Poisson shrinker
